# Sheldon on Congestive Fever

**Published:** 1850-09

**Authors:** Thos. Sheldon

**Affiliations:** Brushy Prairie, LaGrange Co., Ind.


### ARTICLE III.

*Congestive Fever.*—By THOS. SHELDON, M. D. Brushy Prairie, LaGrange Co., Ind.

Perhaps there is no more vague term used in Medicine, than the one at the head of this communication. By it I wish to designate a form of Fever, with which I have frequently met in practice, but a description of which I have never been so fortunate as to meet with; consequently what I write is the result of my own observation at the bed-side of the patient.

I should define this disease to be, Continued Fever of rather a low grade of excitement, with obstinate inaction of the biliary organs; owing probably to congestion in the system of the vena portarum. It may occur primarily, or may follow in the course of, or upon the subsidence of other diseases, more especially Pneumonia.

When the disease occurs as a primary affection, the premonitory symptoms do not differ from those of common Bilious Fever; in fact, the full developement of the disease is frequently preceded by Fever of an Intermittent form. There is chilliness, sometimes a distinct chill, languor, pain in the head, back, and extremities, vertigo, loss of appetite, thirst, sometimes extreme restlessness, oftener, drowsiness, or inclination to stupor; bowels obstinately constipated, or harrassed by a colliquative diarrhœa; the tongue is dry in the centre, red at the tip and edges, becoming dark colored and cracked in the course of the disease; frequently the point has a peculiarly smooth and shiny appearance. Sometimes constant nausea and vomiting without the ejection of any bile; the skin and conjunctiva are yellow, and the urine scanty and high colored. The pulse is generally frequent and compressible, sometimes very much oppressed, making the feel of it at the wrist difficult and indistinct. When the oppression is great, the breathing will be irregular and the skin cool; the pulse perhaps not perceptible at the wrist. This condition is only distinguishable from one of prostration by the preceding history of the case. Internal stimulants under these circumstances will prove, so far as my experience goes, injurious. The sleep is generally disturbed, the patient starting and scowling or muttering in his sleep. The skin may be dry or moist; the extremities inclined to be cool.

Cases do occur in which the patient complains of no pain whatever, through the whole course of the disease; neither is there tenderness or pressure over the thoracic or any part of the abdominal regions. Delirium usually supervenes when the disease goes on toward an unfavorable termination.

The characteristic symptom, I conceived to be, the appearance of the alvine discharges, and their not being changed by the administration of ordinary bilious cathartics. The stools are extremely thin, watery, without much fœtor, and continue thus, even when calomel and other mercurial cathartics have been freely administered. When the mercurials have been continued sometime in moderate quantities, the stools assume a greenish tinge, but still continue scanty and without consistence. So long as the stools retain this appearance the Fever continues unabated.

Remissions and exacerbations frequently occur, the remission in the morning, the exacerbation in the afternoon or evening; but the remission is imperfect, merely a diminution of of the febrile excitement which is at no time excessive.

When the disease goes on to a fatal termination, the stupor increases, or when restlessness prevails, it continues unabated; the tongue becomes dryer and darker in the centre, more red and shiny at the edges; the pulse quicker, more frequent, and irregular; the teeth are covered with dark sordes; the countenance assumes an anxious expression, and toward the close becomes cadaverous; the breathing irregular; delirium and subsultus tendinum ensue; the extremities become cold; the skin clammy; the pulse weaker, more frequent, and irregular, and death soon closes the scene.

When the disease yields to the efforts of nature, or to the influence of remedies, the stupor diminishes; the tongue becomes cleaner, moister, and less red at the tip and edge; the pulse fuller and slower; the skin uniformly warm and moist, and, which is of greater importance, the stools become free, consistent and dark colored; the appetite returns and the patient is convalescent. I have not met with any case in which the stools assumed these appearances, which did not terminate favorably; but I have seen marked improvement in all other respects, which was merely temporary, and was soon followed by an aggravation of all the symptoms and a fatal termination.

When this condition supervenes upon the subsidence of Pneumonia, the Pneumonic symptoms yield, but the patient does not improve as rapidly as we naturally anticipate; the bowels are not moved by mild cathartics, or colliquative diarrhœa is present; the tongue becomes dark colored at the base and centre, and red and shiny at the tip and edges; the skin and conjunctiva are yellow, the pulse quick and frequent; and unless speedily interrupted, the train of symptoms before enumerated, terminating in death, follow in quick succession. I have witnessed this result several times in persons of intemperate habits, who had been severely attacked with Pneumonia, for which they had been actively treated in the onset. These cases I believe to be peculiarly unmanageable, owing to the impaired condition of the biliary organs, in intemperate persons.

During the past season this form of Fever prevailed extensively in this neighborhood, and was very fatal, The want of success which attended my practice while pursuing an ordinary course of treatment, led, or rather forced me to adopt the one which I am about to detail, and which so far as I have seen it tried has proved entirely successful.

I will merely remark in regard to my former practice and experience in this disease, that I had used bleeding when the pulse or other symptoms called for it; Calomel in full doses, or in small ones repeated; cathartics of every description in different cases; epispastics to the right hypochondrium and over the abdomen; sinapisms and warmth to the extremities; opiates, diaphoretics, &c., in short pursuing an ordinary course of treatment for continued Fever with portal torpor or congestion. I gave quinine where the remission was at all distinct. In those cases where I gave calomel in full doses, say from 40 to 80 grs. pr diem, the progress of the disease was retarded, but in a majority of cases, only retarded, the fatal termination though delayed, almost uniformly ensued. Under the use of non-mercurial cathartics, and other courses of treatment, including the expectant plan, which I tried, the success was about the same, but the disease progressed more rapidly than under the former one.

I hold that the indication of cure, is to promote a free action in the system of the vena portæ, which will be evinced by the appearance of free, consistent, dark colored stools. The only remedy I have found capable of effecting this indication, is calomel; and it only when given in enormous doses. In a case of this kind of Fever, if the symptoms are not very prominent, or alarming, or such as to leave no doubt of its character, I administer an ordinary purgative dose of Calomel, say from 10 to 40 grs. followed by some other cathartic; if the stools produced by this are free and consistent, we have the best evidence that portal congestion is not present; but if on the contrary, the stools continue thin and watery, and the fever not materially abated, I hesitate no longer, but administer (generally with my own hands,) from one to two hundred grs. Calomel, and repeat it according to the severity of the attack, in from 40 to 120 grs. doses, every 3 or 4 hours, until the passages become free and consistent. After from 12 to 18 hours if the Calomel does not operate freely, I prescribe some additional cathartic medicine; a spoonful of castor oil with a drop or two of croton oil repeated every 2 hours answers very well, where it will be retained by the stomach.

When other articles are rejected, the castor oil in pill, aided by stimulating enemata, will be most efficient, When there are evidences of mucous irritation in the intestinal canal, I prefer a mixture of Castor Oil and Magnesia, flavored by Oil of Lemon or Peppermint.

The Calomel should not be discontinued until the stools are *free*, *consistent*, and *dark colored*; neither should opium be combined wi[illegible] it to prevent its running off by the bowels.

The object is, to open all the secretions, not to get the specific effect of the mercury, and no danger of this need be apprehended, unless the Calomel is continued after the above named indication is entirely fulfilled.

At the same time if there is much oppression, with stupor, coldness of the extremities etc. I apply a large epispastic to the right hypochondrium, sinapisms and warmth to the extremities, and give the spiritus mindereri internally.

The size of the dose of Calomel, and the frequency of its repetition, must of course depend upon the severity of the attack, and the time which has elapsed since its invasion. The earlier in the disease the Calomel is administered, the more prompt will be its effect, and the less of it will be required to fulfil the indication.

To one person who was most severely attacked I gave 200 grs. Calomel at one dose, followed in 6 hours by Croton Oil; he took 5 drops of the Oil, was purged very freely, the stools, which were at first extremely thin and watery, became consistent, and he convalesced rapidly without the slightest appearance of ptyalism. The same individual, about six months previously, was salivated by taking 20 grs. Calomel followed by Sulph. Magnesia, which also operated freely, for an ordinary attack of Bilious Fever.

In another case, one of the most alarming ones I ever saw, I gave 360 grs. Calomel in the course of the day; Castor Oil and Magnesia were given, which operated during the night; the following morning there was a striking improvement in all the symptoms; I gave him 120 grs. more at one dose, and the following day he was convalescent, without ptyalism. This patient was a young man of very feeble constitution, and depraved general health.

To a little girl 6 years of age, who had been laboring under the disease several days, and had been taking mercurials and Cathartics, Diaphoretics, Quinine, etc., without any perceptible improvement, I gave 180 grs. in divided doses within 12 hours; this followed by the Oil and Magnesia mixture, produced two very copious stools, a considerable portion of which was of the consistence of Tar and very dark colored. From this time she convalesced rapidly, without ptyalism; and her constitution which was previously good, is in nowise impaired by the enormous doses of this powerful remedial agent.

The Calomel is the principal remedy, but the Spts. Mindereri, especially when the disease has continued sometime I hold of very great importance. As a laxative the oil and Magnesia mixture is the best that can be used.

The use of Opium should as much as possible be avoided; and when given, very small doses will be found most beneficial; a little Comp. Tinct. Opii will almost always allay any irritation that may arise. So long as the stools are thin and watery, Opium will be *positively injurious.*

When abdominal tenderness supervenes, the application of cold by means of linen cloths dipped in cold water, and applied over the whole abdomen, changing them as often as they become warm, will be found very serviceable.

Where Dysury is present, the Spts. Nit. Dulc., together with Slippery Elm infusion, may be given with advantage.

After a free action of the biliary organs has been obtained, some of the Bitter Infusions will hasten the complete restoration of health. Quinine will be necessary when Fever of a periodic character is present. But until all the secretions are fully established, the administration of stimulants or tonics cannot be otherwise than injurious.

A few words more as to the administration of Calomel in these enormous doses and I will close. I have known of but two instances, out of a considerable number, in which the Calomel was given in quantities of from 2 to 400 grs. in the 24 hours, and in some instances repeated in these doses for several days, in which Ptyalism took place; and in these it was not profuse. In both, the Calomel was continued, though in smaller doses, after the stools had become free and consistent.

No considerable nausea or other uneasy feeling is produced while the Calomel is slowly but surely fulfiling its office. In no case have I seen anything like Hypercatharsis or excessive purgation follow its exhibition. I have frequently regretted not having given it sooner or more freely, but never had a wish that the dose had been less.

Unquestionably Calomel is an agent of great power for good or ill; and its administration, especially in these doses, should not be ventured upon rashly, or trusted to the hands of an unskilful person; but the effect of the remedy should be closely observed, and as soon as the desired effect is obtained, but not before, it should be discontinued. It can be most conveniently given by mixing with sweet cream.

Very probably a less energetic course of treatment will sometimes prove successful; but cases will occur in which I am very confident no other remedy will be found equal to the disease.

Epidemics vary greatly, as to severity in different years, and in different places the same year. That which prevailed here the past season was of a very obstinate character; but I can well remember cases which occurred in my practice in former years, terminating fatally, in which I believe a different result would have followed the adoption of this plan of treatment.

I have never known the stage of collapse to ensue, when the secretions were fully restored; neither have I ever known a patient to recover from this form of Fever when it did take place ; consequently, anything I might be able to write concerning this stage would not be useful or interesting.

Stimulants may be given freely, but in all probability will prove entirely unavailing.

An advanced state of oppression might be mistaken for one of prostration ; the former would require the free use of the Calomel, and the latter could hardly be injured by it ; of course, where there was such a doubt existing, I would recommend that course of treatment which would offer the best chance of saving the patient.

